# Supplementary material for: Characterizing the Role of Biologically Relevant Fluid Dynamics on Silver Nanoparticle Dependent Oxidative Stress in Adherent and Suspension In Vitro Models
Source: Antioxidants (Basel). 2021 May 23;10(6):832. doi: 10.3390/antiox10060832 (PMC8224783; doi:10.3390/antiox10060832)
Supplement: Supplementary file 1 [file antioxidants-10-00832-s001.zip › antioxidants-1230402-supplementary.pdf]

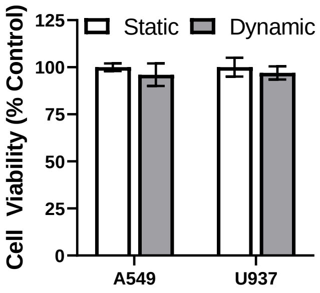

Supplementary Figure 1: Cytotoxicity Dynamic Controls. Following a 24 hour within either (A) A549 or (B) U937 models the cellular viability was assessed. These results indicated that within both systems no changes in viability were denoted as a function of flow. * indicate statistical significance from the untreated control, n=3, p<0.05.

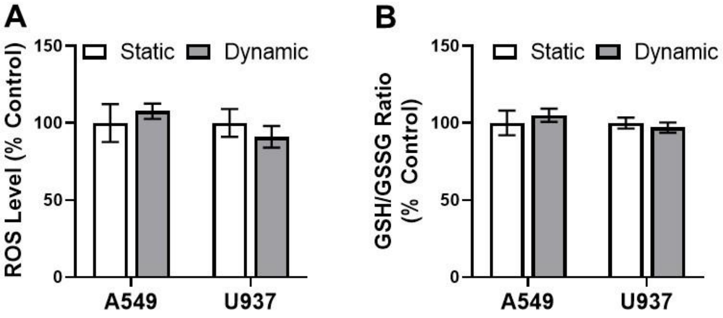

Supplementary Figure 2: Oxidative Stress Dynamic Controls. Following a 24 hour within either A549 or U937 models (A) ROS and (B) GSH/GSSG ratios were quantified. These results indicated that within both systems no changes in intracellular oxidative stress markers were denoted as a function of flow. * indicate statistical significance from the untreated control, n=3, p<0.05.

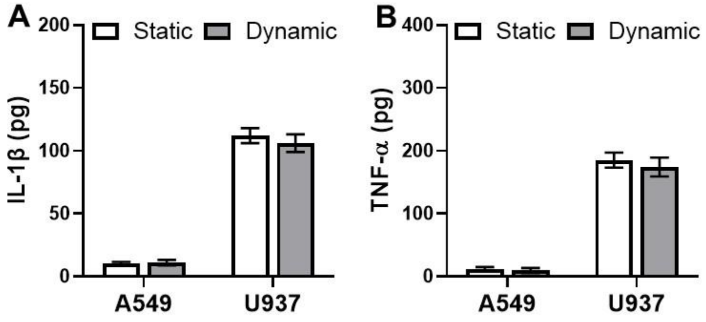

Supplementary Figure 3: Cytokine Secretion Dynamic Controls. Following a 24 hour within either A549 or U937 models the levels of extracellular (A) IL-1β and (B) TNF-α were measured. * and indicate statistical significance from the untreated control, n=3, p<0.05.
